# Development of a Direct Cell-to-PCR Lysis Buffer Using Optimized Non-Ionic Detergents for RNA-Extraction-Free RT-qPCR

**DOI:** 10.3390/mps9040114

**Published:** 2026-07-31

**Authors:** Mahmoud Zhra, Rawan Awni Alarawi, Shaimaa Abdelrahman Mohamed, Abdel Naser Daoud, Hana Fakhoury, Ahmad Aljada

**Affiliations:** 1 Department of Biochemistry and Molecular Medicine, College of Medicine, Alfaisal University, Riyadh 11533, Saudi Arabia; mzahra@alfaisal.edu (M.Z.); ralarawi@alfaisal.edu (R.A.A.); 2 College of Medicine, Alfaisal University, Riyadh 11533, Saudi Arabia; shmohamed@alfaisal.edu; 3 Department of Mechanical Engineering, College of Engineering, Alfaisal University, Riyadh 11533, Saudi Arabia; adaoud@alfaisal.edu

**Keywords:** direct lysis, RT-qPCR, RNA extraction-free workflow, non-ionic detergents, low-input gene expression, single-cell detection, clinical swabs, SARS-CoV-2

## Abstract

Column-based RNA extraction is time-consuming, involves cumulative losses during transfer, wash, and elution steps, and has reduced recovery from sub-microliter inputs. These limitations restrict gene-expression analysis in small cell populations and low-titre pathogen detection in clinical specimens. We developed the Direct Cell-to-PCR Lysis Buffer, a defined non-ionic detergent formulation containing Tween 20 (0.3%), Triton X-100 (0.1%), and NP-40 (0.1%), for RNA-extraction-free one-step RT-qPCR. Lysates are added directly to reactions without pretreatment, heating, or column purification. Non-ionic detergents produced Ct values comparable to detergent-free controls, whereas SDS, Sarkosyl, and sodium deoxycholate abolished amplification. The method was benchmarked against the standard column-based PureLink RNA Mini Kit using SaOS-2 cells, MCF7 cells, peripheral blood mononuclear cells, and nasal and throat swabs. Direct lysates showed Ct offsets of 0.6 to 2.85 cycles compared with purified RNA, depending on sample type. Ubiquitin-C mRNA was detected down to a single-cell-equivalent input, and plasmid templates were detected to approximately 100 copies per reaction. The buffer was compatible with multiplex ScriptTaq COVID PCR, with unchanged RdRP, N, and RPP30 Ct values, and tolerated sodium azide up to 0.1%, supporting rapid direct RT-qPCR workflows.

## 1. Introduction

The recovery of RNA from small or rare cell populations remains a major limitation in gene-expression analysis and in the molecular diagnosis of low-titre infections. This is particularly relevant to circulating tumor cell biology, rare-pathogen detection, single-cell analysis, and small clinical specimens, where limited input material increases the risk of RNA loss and compromises downstream reverse transcription quantitative PCR (RT-qPCR) or RNA sequencing (RNA-Seq).

Column-based RNA extraction remains the standard approach in many laboratories. These workflows rely on solid-phase capture, where nucleic acids bind to silica membranes and are eluted after multiple wash steps. Although robust and widely used, they involve repeated transfers, washes, and elution steps that can reduce RNA recovery, especially from sub-microliter or low-input samples. Residual genomic DNA carry-over can also affect transcript quantification and may generate misleading positive signals in assays targeting low-abundance templates. These limitations have driven the development of alternative extraction and sample-preparation methods.

Several improved RNA-recovery approaches have been reported, including two-phase washing to reduce buffer carry-over [1], chitosan-coated silica [2], porous polymer monoliths [3], centrifugation-assisted immiscible-fluid filtration [4], and surface-tension-valve cassettes [5]. These methods address different needs, including automation, low-resource testing, rare-cell recovery, and improved yield. However, each approach involves trade-offs in cost, complexity, input volume, throughput, and compatibility with downstream assays [6,7].

Direct lysis provides an alternative strategy by bypassing purification. In this approach, cells are lysed in a buffer compatible with reverse transcription, and an aliquot of crude lysate is added directly to RT-qPCR or cDNA synthesis. Removing wash and elution steps reduces handling and potential RNA loss. Viet-Phuong Le et al. showed that direct lysis can outperform column-based extraction at low cell input, with detectable signal from as few as ten cells per reaction [8]. Similar workflows have been applied to gene-expression assays, three-dimensional culture systems, and SARS-CoV-2 diagnostics [8,9,10], where extraction-free workflows shortened turnaround time during high-volume testing [11,12]. These studies show that direct lysis can improve practicality, although performance remains dependent on sample type and assay conditions [13,14,15].

Commercial direct-lysis systems are available, including the SuperScript Direct Cell Lysis and RT Kit, the Single Cell-to-CT qRT-PCR Kit, and QuickExtract RNA Extraction Solution. These products support direct RT-qPCR workflows, but their cost and limited lysate-input tolerance may restrict use in high-throughput settings. QuickExtract RNA Extraction Solution accepts 1–10 µL of input directly into a 20 µL reverse-transcriptase reaction [16], and detergent-mediated viral inactivation has been characterized independently for hepatitis C and Ebola viruses [17,18].

Here, we describe an optimized Direct Cell-to-PCR Lysis Buffer based on non-ionic detergents for RNA-extraction-free one-step RT-qPCR. The study evaluates detergent compatibility, buffer formulation, performance across cell lines, PBMCs, and clinical swabs, low-input sensitivity, multiplex SARS-CoV-2 RT-qPCR compatibility, and sodium azide tolerance. Throughout, the Direct Cell-to-PCR Lysis Buffer is benchmarked directly against the current standard method, column-based silica-membrane RNA extraction (PureLink RNA Mini Kit), across all cultured-cell and clinical sample types, with paired cycle-threshold (Ct) comparisons reported for every matrix and across template-input and polymerase titrations.

## 2. Materials and Methods

### 2.1. Materials

Sodium dodecyl sulfate (SDS; Cat. No. PR0611) was obtained from Vivantis Technologies, Subang Jaya, Selangor, Malaysia. Tergitol type NP-40 (NP-40; nonylphenoxypolyethoxyethanol, 70% in H_2_O; Cat. No. NP40S), Tergitol type NP-9 (NP-9; nonylphenoxypolyethoxyethanol; Cat. No. NP9), sodium *N*-lauroylsarcosinate (Sarkosyl; Cat. No. 61739), polyoxyethylene (20) sorbitan monolaurate (Tween-20; Cat. No. P9416), octylphenol ethoxylate (Triton X-100; Cat. No. T8787), and sodium azide (Cat. No. 71289) were purchased from Sigma-Aldrich, St. Louis, MO 63103, USA. Sodium deoxycholate (Cat. No. 89905) was obtained from Fisher Scientific, Hampton, NH, USA. Nuclease-free water was used for all buffer preparations.

### 2.2. Preparation of Direct Cell-to-PCR Lysis Buffer

The Direct Cell-to-PCR lysis buffer was prepared in nuclease-free water to final concentrations of 0.3% (*v*/*v*) Tween-20, 0.1% (*v*/*v*) Triton X-100, 0.1% (*v*/*v*) NP-40, and 0.03% (*w*/*v*) sodium azide as a preservative. The buffer was mixed by vortexing until homogeneous, aliquoted into 10 mL tubes, and stored at 4 °C for several weeks.

### 2.3. Direct Cell-to-PCR Sample Lysis and RT-qPCR Workflow

For each sample type, lysates were prepared using the Direct Cell-to-PCR lysis buffer and used directly as templates for one-step RT-qPCR without RNA purification.

#### 2.3.1. Cultured Cell Lysates: SaOS-2 and MCF7 Cells

SaOS-2 cells (ATCC HTB-85) and MCF7 cells (ATCC HTB-22) were cultured in DMEM supplemented with 10% fetal bovine serum and 1% penicillin–streptomycin at 37 °C in a humidified incubator with 5% CO_2_. Cells were harvested at approximately 80–90% confluency using trypsin, pelleted at 200× *g* for 5 min, and resuspended in 200 µL of either Direct Cell-to-PCR lysis buffer or the lysis solution supplied with the PureLink RNA Mini Kit, using the manufacturer-recommended volume. The lysates were mixed thoroughly by vortexing until homogeneous and then used directly for RT-qPCR. Serial dilutions of cell lysates were also prepared in Direct Cell-to-PCR lysis buffer prior to RT-qPCR, with dilution continued down to the single-cell equivalent.

#### 2.3.2. PBMC Lysates

Peripheral blood mononuclear cells (PBMCs) were isolated by Ficoll-Paque density gradient centrifugation. Whole blood was collected in EDTA tubes and layered onto an equal volume of Ficoll-Paque solution (density 1.077 g/mL; GE Healthcare Bio-Sciences AB, Uppsala, Sweden). Samples were centrifuged at 400× *g* for 30 min at room temperature with the brake off. The PBMC layer was collected, washed twice with PBS, and resuspended in 200 µL of either Direct Cell-to-PCR lysis buffer or the lysis solution supplied with the PureLink RNA Mini Kit (Thermo Fisher, Waltham, MA, USA), using the manufacturer-recommended volume.

The lysates were mixed thoroughly by vortexing until homogeneous. A portion of each sample was processed according to the PureLink RNA Mini Kit protocol, whereas the remaining portion, suspended in Direct Cell-to-PCR lysis buffer, was used directly for RT-qPCR.

#### 2.3.3. Clinical Nasal/Throat Swab Lysates

Clinical nasal and throat swab specimens were collected in 200 µL of either Direct Cell-to-PCR lysis buffer or the lysis solution supplied with the PureLink RNA Mini Kit. The specimens were mixed thoroughly by vortexing until homogeneous and then processed as described above. Samples collected in the PureLink RNA Mini Kit lysis solution were processed according to the manufacturer’s protocol, whereas samples collected in Direct Cell-to-PCR lysis buffer were used directly for RT-qPCR.

#### 2.3.4. Template Input

Unless otherwise specified, RT-qPCR reactions were assembled in a final volume of 20 µL. For Direct Cell-to-PCR reactions, lysate input volumes of 2.5, 5, 7.5, and 10 µL were evaluated. Based on these evaluations, a maximum lysate input volume of ≤5 µL was recommended to minimize potential PCR inhibition, particularly in sensitive sample matrices.

### 2.4. Column-Based RNA Isolation

RNA was isolated using the PureLink RNA Mini Kit (Thermo Fisher; Cat. No. 12183020) according to the manufacturer’s protocol. Purified RNA was eluted in 50 µL of RNase-free water supplied with the kit.

### 2.5. Ubiquitin-C (UBC) One-Step RT-qPCR Assay

UBC RT-qPCR was performed using a probe-based one-step format. The final primer and probe concentrations were 0.4 µM and 0.2 µM, respectively. The oligonucleotide sequences were as follows: forward primer, 5′-ACTACAACATCCAGAAAGAGTCCA-3′; reverse primer, 5′-CCAGTCAGGGTCTTCACGAAG-3′; and probe, 5′-6-FAM-CCCACCTCTGAGACGGAGCACCAG-BHQ-1-3′.

Reactions were run on a Bio-Rad CFX96 system under the following cycling conditions: reverse transcription at 50 °C for 10 min; initial denaturation at 95 °C for 5 min; followed by 40 cycles of denaturation at 95 °C for 15 s and annealing/extension at 58 °C for 45 s.

### 2.6. Multiplex SARS-CoV-2 RT-qPCR

An in-house multiplex real-time RT-qPCR assay (ScriptTaq COVID PCR) targeting SARS-CoV-2 RdRP and N genes (WHO/CDC-published sequences) and human RPP30 as a sample quality control was used to test lysis buffer compatibility. Positive controls included plasmid clones pUC57-2019-nCoV-PC: RdRP and pUC57-2019-nCoV-PC: N (GenScript, Piscataway, NJ, USA) and an RPP30 control (Rockville, MD, USA). Primer/probe sequences are listed in Table 1. The assay was previously validated to detect down to 100 copies per reaction for RdRP and N targets [19].

### 2.7. Statistical Analysis

Statistical analysis was performed using SigmaStat version 3.5. Data are reported as mean ± SEM, and the number of replicates (*n*) is stated in each table legend. The detergent-compatibility and titration screens (Table 2 and Table 3) used a single reaction per condition and are reported descriptively. Replicates throughout are technical. For each condition, a single lysate or RNA preparation was divided across three RT-qPCR reactions. The reported dispersion therefore reflects analytical variability within a preparation, not variation between independent biological samples. For the technical-triplicate benchmarking of Direct Cell-to-PCR lysate against column-purified RNA, two-group comparisons at a matched condition were made using Student’s *t*-test, with the paired or unpaired form selected according to the experimental design. The two-factor titrations, in which method was compared across input volume (Table 5) and Taq-polymerase concentration (Table 6), were analyzed by two-way analysis of variance (ANOVA) with the Holm–Šidák correction for multiple comparisons. Linearity of the plasmid and cell-dilution series was assessed by linear regression of Ct on log10 template. Differences were considered statistically significant at *p* < 0.05. The consolidated quantitative data were plotted using GraphPad Prism V8 (GraphPad Software, San Diego, CA, USA).

## 3. Results

### 3.1. Detergent Compatibility and Titration Using UBC RT-qPCR

We assessed the compatibility of commonly used ionic and non-ionic detergents with one-step RT-qPCR by measuring UBC Ct values relative to a detergent-free control. Detergents were tested at a final concentration of 0.025% (*w*/*v* or *v*/*v* as applicable) in reactions containing purified RNA from MCF7 cells (Table 2). Ionic detergents (SDS, Sarkosyl, and sodium deoxycholate) completely inhibited amplification under the tested conditions (Ct Undetermined). In contrast, non-ionic detergents (Tween-20, Triton X-100, NP-9, and NP-40) did not inhibit amplification at 0.025%, producing Ct values comparable to the no-detergent control.

**Figure 1:**
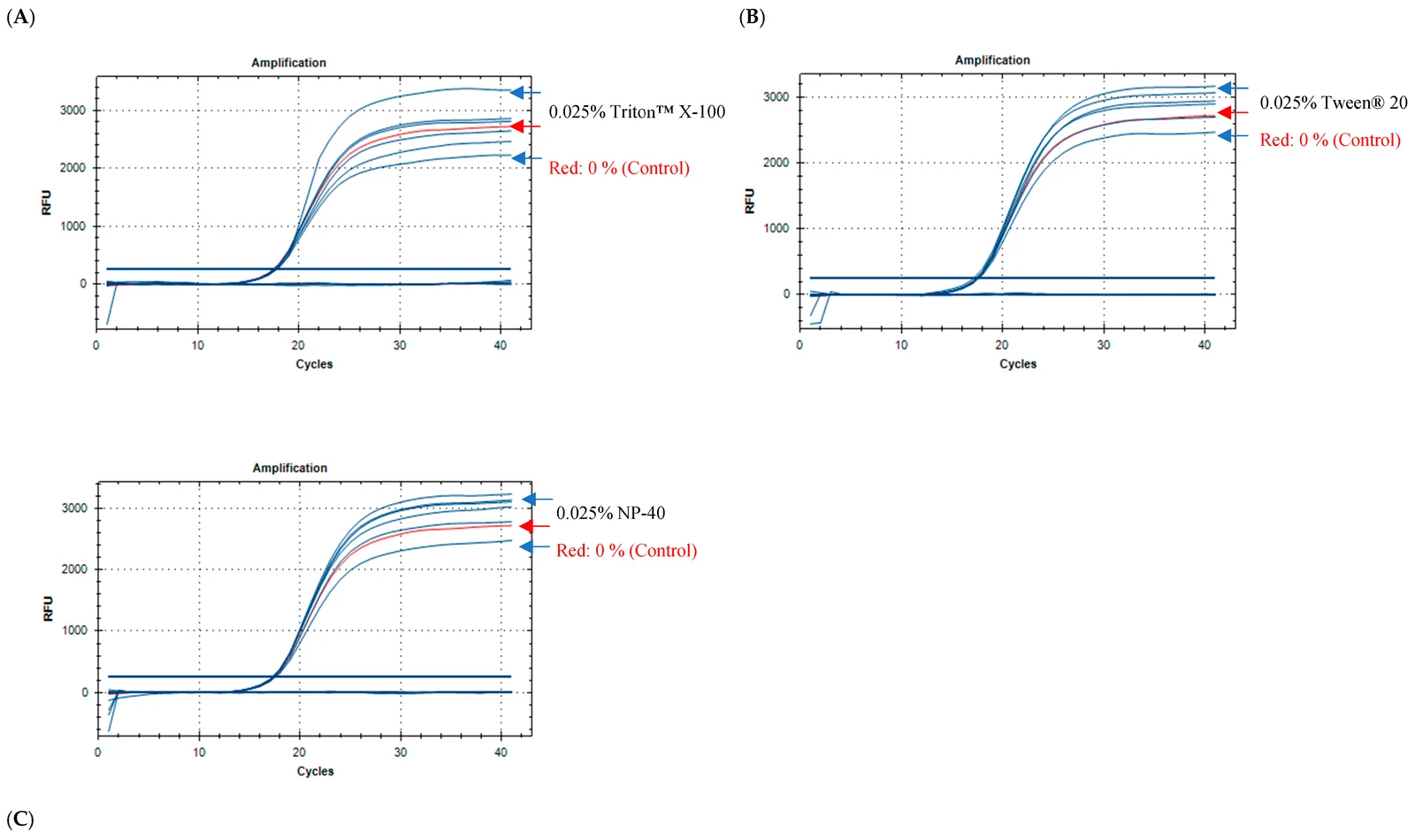
Effect of non-ionic detergents on RT-qPCR amplification curves. UBC RT-qPCR was performed using purified RNA from MCF7 cells in the absence or presence of varying concentrations of non-ionic detergents. Amplification curves are shown for Triton X-100 (**A**), Tween 20 (**B**), and NP-40 (**C**).

To identify concentrations compatible with RT-qPCR while preserving lysis capability, Triton X-100, Tween-20, and NP-40 were titrated from 0.5% to 0.025% using purified RNA from MCF7 cells (Table 3). Across the tested range, Ct values showed only minor variation relative to the detergent-free control. At higher detergent concentrations, amplification curve fluorescence intensity decreased, whereas lower concentrations yielded higher fluorescence intensity (Figure 1).

### 3.2. Selection of the Direct Cell-to-PCR Lysis Buffer Formulation

Based on detergent compatibility testing and titration experiments, we selected a combined non-ionic detergent formulation containing 0.3% Tween-20, 0.1% Triton X-100, and 0.1% NP-40, with 0.03% sodium azide added as a preservative. These concentrations were chosen to balance RT-qPCR compatibility with lysis performance, informed by our titration experiments and prior reports evaluating lysis efficiency and detergent-mediated viral inactivation when used separately [20,21]. When added to UBC RT-qPCR reactions containing purified RNA from MCF7 cells, the combined formulation produced Ct values comparable to the no-buffer control (Ct 17.32 vs. 17.28) and resulted in a modest increase in fluorescence intensity under the tested conditions.

### 3.3. Benchmarking the Direct Cell-to-PCR Lysis Buffer Against Standard Column-Based RNA Extraction Across Sample Types

We evaluated the performance of the Direct Cell-to-PCR lysis buffer across multiple biological matrices and compared UBC RT-qPCR Ct values obtained from direct lysates versus column-purified RNA isolated with the standard PureLink RNA Mini Kit. Sample types included SaOS-2 and MCF7 cultured cells, PBMCs, and clinical throat and nasal swabs (Table 4). Across matrices, direct lysates yielded Ct values that were shifted by 0.6 to 2.85 cycles relative to column-purified RNA, with the magnitude of the shift depending on sample type. The largest Ct shifts were observed in clinical swab specimens, consistent with matrix-associated inhibition or dilution effects in crude lysates.

### 3.4. Effect of Template Input Volume on Direct Cell-to-PCR Performance

To investigate whether inhibition was dependent on template input volume, we compared Ct values obtained using increasing volumes of either isolated RNA or Direct Cell-to-PCR lysate in a fixed 20 µL UBC RT-qPCR reaction (Table 5). SaOS-2 samples were standardized to 500 cells/µL prior to preparation of lysates or RNA extraction. Across the tested range (2.5–10 µL template input), isolated RNA maintained consistent Ct values. In contrast, Direct Cell-to-PCR lysates showed the greatest Ct delay at higher input volumes, with 10 µL lysate producing an ~2–3 cycle increase relative to isolated RNA, consistent with inhibitor carryover at higher lysate fractions. To determine whether increasing Taq polymerase concentration could reduce the Ct increase observed with direct lysates, we evaluated the effect of Taq polymerase concentration on amplification performance (Table 6).

### 3.5. Evaluation of UBC Detection Across Varying Cell Numbers Using Direct Cell-to-PCR Lysates

To assess whether the Direct Cell-to-PCR lysis buffer supports low-input gene expression analysis, UBC amplification was evaluated using serially diluted SaOS-2 cells ranging from 5000 cells to 1 cell per reaction (Table 7). Serial dilutions were prepared and lysed using the Direct Cell-to-PCR lysis buffer, and the resulting lysates were used directly as templates for one-step RT-qPCR without RNA purification. For all tested dilutions, the template input volume was standardized at 5 µL, and the total UBC RT-qPCR reaction volume was 20 µL.

UBC was consistently detected across the full dilution series, with Ct values increasing as cell input decreased, as expected. Notably, UBC amplification was detected down to the single-cell level under the tested conditions, supporting the suitability of this workflow for low-input RT-qPCR applications.

### 3.6. Sensitivity of UBC Detection Across Varying Copy Numbers

To assess analytical sensitivity independent of cellular lysis, a UBC amplicon cloned into a pUC57 plasmid (Bio Basic Inc., Markham, ON, Canada) was tested across a dilution series from 1,000,000 down to 1 copy per reaction (Table 8). Three technical replicates (*n* = 3) were performed for each condition, and Ct values were recorded.

**Figure 2:**
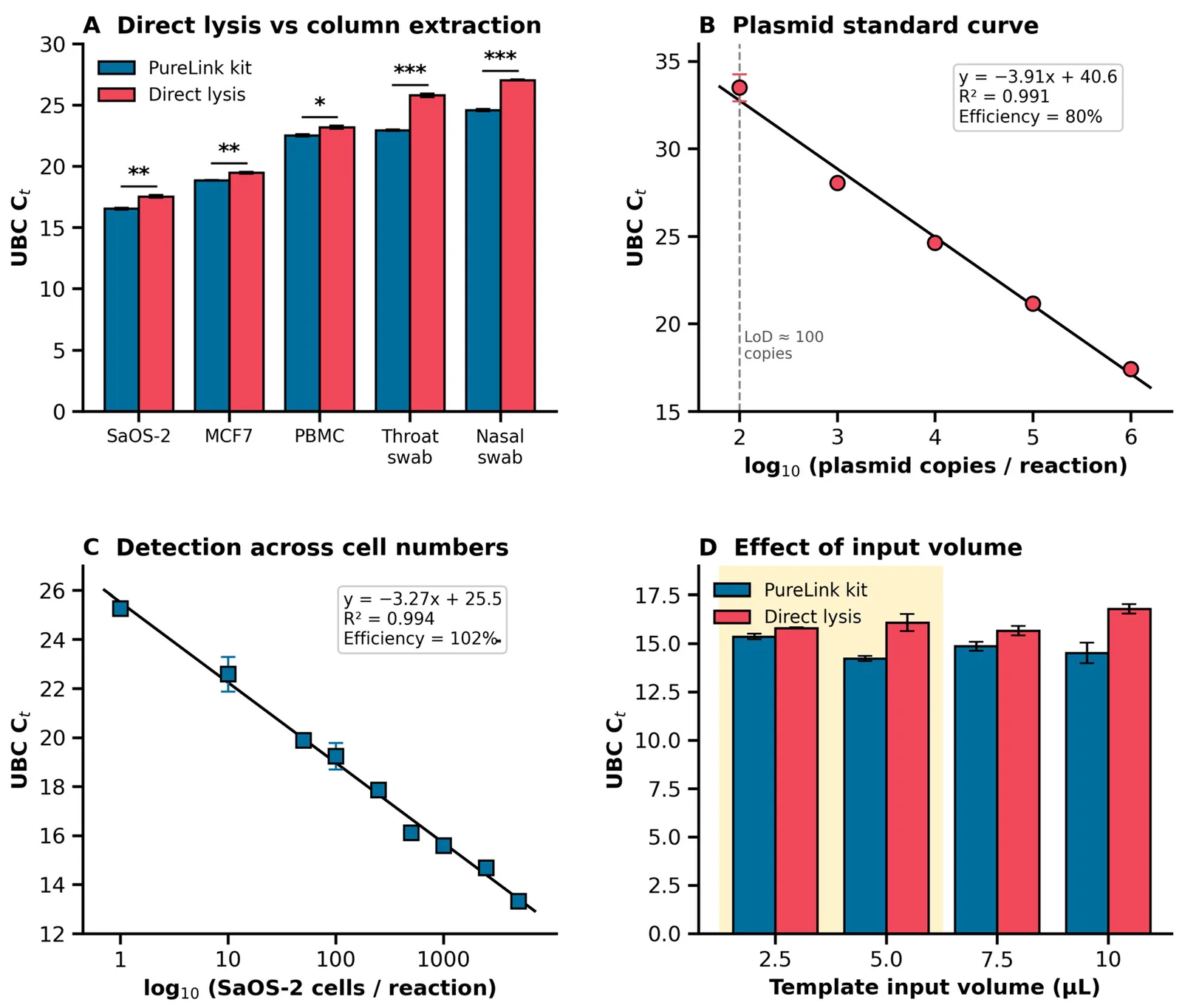
Consolidated quantitative performance of the Direct Cell-to-PCR Lysis Buffer. (**A**) UBC Ct of direct lysates versus column-purified RNA across five sample matrices (mean ± SEM, *n* = 3 technical replicates; significance from unpaired *t*-tests: *, *p* < 0.05; **, *p* < 0.01; ***, *p* < 0.001). (**B**) Plasmid copy-number standard curve. (**C**) UBC detection across a SaOS-2 cell-dilution series. (**D**) Effect of template input volume; the recommended ≤5 µL input range is shaded.

UBC amplification was observed down to 100 copies per reaction under the tested conditions. Reactions containing 10 or 1 copy per reaction did not amplify within 40 cycles (Ct Undetermined), indicating an approximate detection limit of ~100 copies per reaction for this assay configuration. The complete quantitative characterization of the buffer, spanning sample matrices, cell numbers, and plasmid copy numbers, is summarized in Figure 2.

### 3.7. Effect of Direct Cell-to-PCR Lysis Buffer on Multiplex ScriptTaq COVID RT-qPCR

We evaluated compatibility of the Direct Cell-to-PCR lysis buffer with our in-house multiplex real-time RT-qPCR assay (ScriptTaq COVID PCR) designed for qualitative detection of SARS-CoV-2 RdRP and N targets with human RPP30 as an internal sample control [19]. To isolate the effect of the lysis buffer on amplification performance, plasmid clones pUC57-2019-nCoV-PC: RdRP and pUC57-2019-nCoV-PC: N (GenScript, Piscataway, NJ, USA) and an RPP30 control template (Rockville, MD, USA) were used as positive controls.

**Figure 3:**
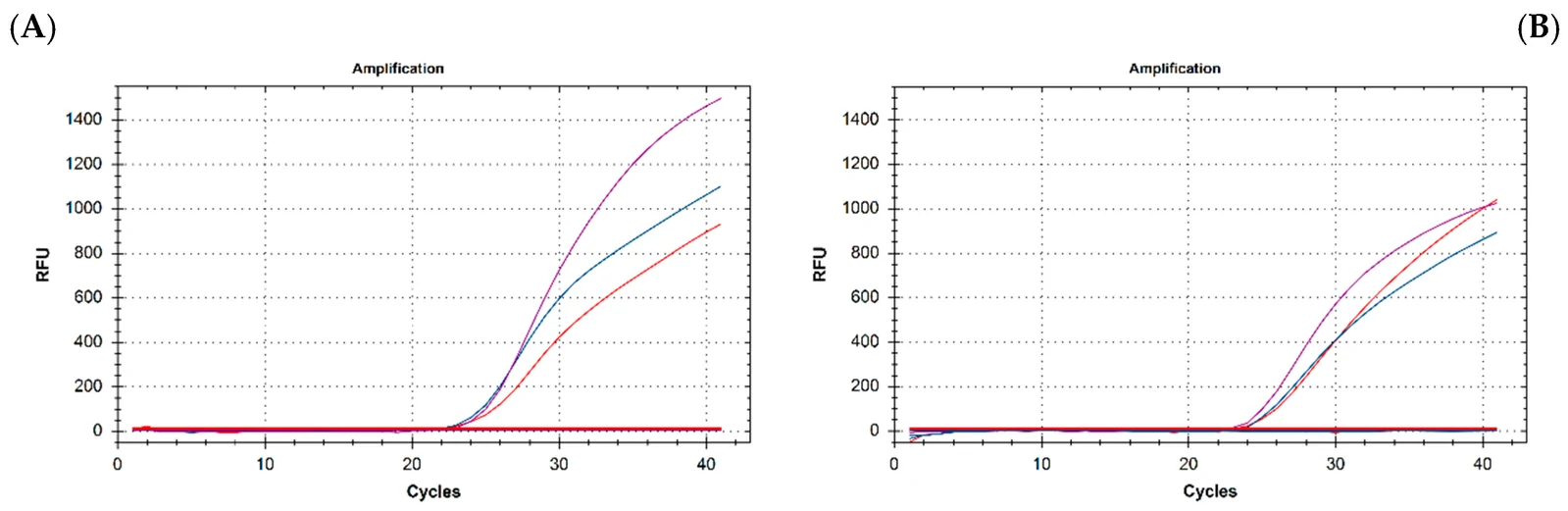
Effect of Direct Cell-to-PCR Lysis Buffer on multiplex SARS-CoV-2 RT-qPCR. Amplification curves from the ScriptTaq COVID PCR assay targeting SARS-CoV-2 RdRP and N, with human RPP30 as an internal control. Reactions were performed without Direct Cell-to-PCR Lysis Buffer (**A**) or with Direct Cell-to-PCR Lysis Buffer (**B**).

In the presence of Direct Cell-to-PCR lysis buffer, Ct values for RdRP, N, and RPP30 were comparable to reactions run without lysis buffer under the tested conditions (Figure 3). Differences were primarily observed in fluorescence intensity rather than Ct, indicating that the buffer did not measurably compromise amplification timing for these targets in this assay configuration.

### 3.8. Effect of Sodium Azide on Multiplex RT-qPCR Performance

Because sodium azide can serve as a preservative for improved reagent stability, we tested whether sodium azide interferes with multiplex RT-qPCR performance. Sodium azide was added to the ScriptTaq COVID PCR assay at final concentrations of 0.01%, 0.03%, 0.05%, and 0.1% and compared with a no-azide control (Table 9). Across all three targets (RdRP, N, and RPP30), Ct values measurements remained similar across azide concentrations, suggesting that sodium azide up to 0.1% does not materially inhibit this multiplex RT-qPCR assay under the tested conditions.

## 4. Discussion

Direct cell-lysis buffers offer an alternative to traditional RNA-isolation protocols that is now competitive on yield, turnaround, and cost. Standard column-based methods couple cell lysis to RNA purification, with successive wash and elution steps that remove proteins, DNA, and lipids but also impose RNA loss at each transfer. The losses matter most when input is limited or rare, and that is precisely where direct lysis has the largest margin: the lysate enters RT-qPCR or cDNA synthesis without further handling, and the workflow gives up nothing on sensitivity at low cell number. Single-cell analyses, CTC preparations, and clinical specimens with low pathogen titres are examples of such applications.

PCR-based assays can quantify low RNA concentrations in crude lysates, often with sensitivity comparable to, or better than, what is achieved after column extraction at minimal cell input [8]. In a clinical workflow, dropping the purification stage cuts the time from sample to result, and turnaround for both infection diagnosis and biomarker monitoring is correspondingly shorter. The same simplification opens the door to high-throughput automation. Population-level genomic studies and drug-screening pipelines stand to gain from this. The advantage is most visible when sample material is itself the constraint, as in CTC cancer studies, infectious-disease diagnostics from minimal volumes, and pediatric or biopsy specimens, where every transfer that loses nucleic acid imposes a real downstream cost.

Designing a direct-lysis buffer for one-step RT-qPCR brings several formulation requirements into play. The buffer has to break the cells open, free the RNA, and shut down any ribonucleases (RNases) before they chew through the template, all without leaving anything behind that would block reverse transcription or amplification. Cutting out organic solvents, centrifugation, and binding/wash steps reduces the protocol steps, and at the same time it eliminates the steps where most RNA loss occurs. The chemistry that delivers all of this in one mixture is constrained by what is compatible with downstream enzymes, which is the principal reason ionic detergents are excluded.

Recent advances in RNA-extraction methodology have substantially expanded the analytic reach of small-population gene expression and low-titre pathogen detection. Direct lysis, solid-phase extraction with porous polymer monoliths, and paper-based platforms address shortcomings of conventional column-based methods in different application niches. Single-cell analysis, biofilm studies, and circulating-biomarker detection are among the fields where these methods deliver the largest analytic gain. As molecular diagnostics and personalized medicine continue to expand, the demand for efficient and reliable RNA recovery will continue to drive method development. Optimizing the recovery step improves the precision and sensitivity of every downstream gene-expression analysis, with direct consequences for disease identification, monitoring, and treatment selection. The cost of commercial direct RT-qPCR lysis buffers nevertheless remains a barrier to wider adoption, and the Direct Cell-to-PCR Lysis Buffer presented here offers a cost-effective alternative that preserves analytic performance, with applications in both research and clinical-diagnostic contexts.

Several applications follow from these properties. For low-input gene-expression analysis, detection of ubiquitin-C down to a single-cell-equivalent makes the buffer suited to circulating tumor cells, sorted rare-cell populations, needle biopsies, and pediatric specimens, where sample material is the limiting factor and every purification transfer carries a real cost in recovered RNA. For molecular diagnostics, the room-temperature, column-free protocol removes the extraction bottleneck that constrains turnaround and reagent supply during high-volume testing, and its compatibility with a multiplex SARS-CoV-2 assay indicates suitability for respiratory-pathogen screening from nasal and throat swabs. Because the buffer is a defined, inexpensive formulation that is stable at 4 °C for several weeks and requires no centrifugation, heating, or specialized equipment, it is also attractive for decentralized or resource-limited laboratories and for high-throughput automation, where fewer liquid-handling steps translate directly into lower cost and fewer errors. Field deployment would nonetheless require dedicated validation of specimen collection, storage, and cold-chain conditions together with operator training, and the present data should be regarded as laboratory-based evidence of performance rather than a validated point-of-care assay.

The buffer may also have utility in handling potentially infectious clinical material. Non-ionic detergents disrupt lipid bilayers, and for enveloped viruses this compromises envelope integrity and exposes the underlying capsid to inactivating processes. The mechanism is best understood in hepatitis C virus. Triton X-100 disrupts the lipid envelope, after which the capsid becomes susceptible to proteolytic and physical degradation [18]. At 0.1% to 0.5%, both Triton X-100 and NP-40 inactivate SARS-CoV-2, with NP-40 reducing titres by ≥6.5 log10 and leaving no recoverable infectious virus [20,21]. For Ebola virus and for herpes simplex virus type 1, 0.1% Triton X-100 is also effective, but only under low-serum conditions [17]. Two caveats nonetheless apply. First, detergent-mediated inactivation is matrix-dependent: 0.1% Triton X-100 does not inactivate Ebola virus in whole blood or physiological serum [17], an outcome attributed to high lipid and protein loads in serum scavenging surfactant activity. Second, non-enveloped viruses lack a lipid bilayer for non-ionic detergents to disrupt and are unaffected by this chemistry. Importantly, these inactivation data derive from studies of the individual detergents rather than from the combined formulation described here. Moreover, the detergent concentrations reported to achieve complete SARS-CoV-2 inactivation can themselves degrade RNA and raise Ct values with prolonged exposure, so effective viral inactivation and optimal RNA recovery may not coincide. We therefore make no claim that the Direct Cell-to-PCR Lysis Buffer inactivates infectious virus. A direct infectivity assay on the formulated buffer would require propagation of live SARS-CoV-2 under Biosafety Level 3 containment, which was beyond the biosafety capability available for this study; such testing is an essential prerequisite before the buffer could be relied upon for viral inactivation and is proposed as future work for laboratories with the appropriate containment.

### Limitations

A reproducible Ct offset relative to column-purified RNA was observed across every matrix tested. For SaOS-2 cells, MCF7 cells, and PBMCs, offsets fell within 0.6–1.0 cycles. Nasal and throat swabs gave 2.5–2.85 cycles. All offsets were derived from three technical replicates (Table 4; *n* = 3, with SEM at or below 0.16). Detection remained reliable in every case, but the offset is consistent enough that laboratories pursuing absolute quantification should build matrix-matched standard curves rather than transfer values from a column-extracted reference. The larger offset for clinical swabs is most plausibly due to co-purification of low-level RT-qPCR inhibitors in these mucosal matrices.

The quantitative Ct offsets reported here were obtained from three technical replicates per condition, that is, three RT-qPCR reactions from a single lysate or RNA preparation, and were reproducible across five independent sample matrices (SaOS-2 cells, MCF7 cells, PBMCs, and nasal and throat swabs) and across five orthogonal titration series (detergent concentration, template input, polymerase concentration, cell number, and copy number). This design establishes analytical reproducibility and shows that the small, systematic Ct offset recurs across biologically distinct matrices and across independent experimental axes. It does not establish biological reproducibility. Because each condition derives from a single preparation, the reported SEM and *p*-values quantify measurement variability within that preparation rather than variation between independent cultures, donors, or specimens. The offsets should therefore not be read as population-level estimates. Independent biological replication using separate cultures, separate donor preparations, and separate clinical specimens is required before these values are generalized, and it is the priority next step for this method.

For external context, Smyrlaki et al. reported a Ct increase of approximately +6.7 cycles for their heat-inactivated extraction-free RT-qPCR relative to column-extracted RNA, with 98.8% accuracy, 96% sensitivity, and 99.8% specificity on clinical specimens [11]. Smyrlaki et al. validated an extraction-free protocol based on heat inactivation (95 °C, 5 min) before reverse transcription, and separately showed that the RT-qPCR tolerated a single non-ionic detergent at high concentration (5% Triton X-100 or 10% Tween 20) for direct lysis without a heat step. The buffer described here takes a different route: three non-ionic detergents at much lower concentrations (0.3% Tween 20, 0.1% Triton X-100, 0.1% NP-40), no heat step. The two assays are tuned for different applications (extraction-free large-scale viral diagnostics in the case of Smyrlaki et al., low-input gene-expression analysis in the present case), and a head-to-head benchmark on identical clinical samples will be needed to establish the relative performance of the two chemistries directly. That comparison remains to be performed.

Considered across the dimensions most relevant to method selection, the present buffer differs from established direct-lysis approaches in defined ways (summarized in Table 10). Relative to the gene-expression method of Viet-Phuong Le et al., which used a single non-ionic detergent (IGEPAL CA-630, an octylphenol ethoxylate and the accepted substitute for the discontinued Nonidet P-40) supplemented with bovine serum albumin and reported signal from as few as ten cells, the present formulation combines three non-ionic detergents at low concentration without added protein and extends reliable detection to a single-cell-equivalent. Relative to the large-scale diagnostic method of Smyrlaki et al., the present buffer operates at room temperature without a heat step and shows a smaller Ct offset versus column-purified RNA (0.6 to 2.85 versus a median of approximately 6.7 cycles), although the two assays were validated on different specimens and optimized for different purposes. In workflow terms, the direct protocol removes the binding, wash, and elution steps of column extraction, reducing hands-on time from roughly 30 to 60 min to a few minutes and lowering per-sample reagent cost, while using the same one-step RT-qPCR chemistry as the column-based reference. A formal head-to-head comparison on identical specimens with matched primer and probe sets remains the definitive test and is proposed as future work.

Genomic DNA carry-over is intrinsic to direct-lysis approaches and is not addressed by the present formulation. Trace contaminating DNA biases gene-expression analyses and produces erroneous transcript quantification. The standard countermeasures (DNase pre-treatment of the lysate or RNA-specific primer designs) both add complexity and reintroduce the RNA loss that direct lysis was designed to eliminate. A more elegant fix would be incorporation of a thermolabile DNase. Enzymes of this class are described in US Patent 8,551,753 B2: irreversible inactivation at approximately 50 °C over 5 min, with substantial double-stranded DNA specificity retained, makes them well suited to decontamination upstream of RT-qPCR. The same patent further covers direct addition of these DNases to the complete reverse-transcription mixture. Adding the DNase directly to the RT mixture has a particular advantage: template decontamination and DNase inactivation both proceed during the RT incubation, with no separate inactivation step before amplification. The reason this matters is practical: when DNase inactivation is run as a stand-alone step between digestion and PCR, residual enzyme or thermal artifacts often perturb downstream amplification, and the in-RT route sidesteps that problem entirely. Standard RT incubation conditions overlap the inactivation window of these enzymes, which means the existing thermal profile of a one-step RT-qPCR run can accommodate the additional DNase activity without modification of cycling parameters. The remaining unknown is the buffer-specific working point: enzyme dose and incubation time will need to be titrated against the present detergent mixture before complete genomic DNA removal can be achieved alongside intact RNA recovery and full reverse-transcription efficiency. That titration has not been carried out here.

Template input volume influenced Direct Cell-to-PCR performance. Although lysate inputs of up to 10 µL in a 20 µL reaction were evaluated, the 10 µL input produced the largest Ct delay relative to isolated RNA, and increasing Taq polymerase concentration did not fully eliminate this offset. Therefore, routine use of ≤5 µL lysate per 20 µL RT-qPCR reaction is recommended to minimize inhibition, particularly for sensitive sample matrices or low-abundance targets. Higher lysate inputs may be feasible in some assay settings, but they should be validated independently for each enzyme system, target, and sample type.

The QE buffer has been used in one-step lysis protocols feeding directly into RT-qPCR, including the 2019-nCoV assay [22]; detergent-mediated viral inactivation in those workflows draws on independent studies of hepatitis C and Ebola viruses, where detergents reduce infectious titres in the absence of serum [17,18].

Detergent selection turns out to be critical for any direct-lysis approach: the ionic detergents tested (SDS, Sarkosyl, sodium deoxycholate) completely inhibited PCR at concentrations as low as 0.025% (Table 2), consistent with their capacity to denature both the reverse transcriptase and Taq polymerase. The non-ionic combination chosen here was guided by three considerations: gentle lysis, compatibility with nucleic acids in RT-qPCR, and prior reports of viral inactivation. Triton X-100 at 0.1–0.5% has been shown to produce substantial reductions in SARS-CoV-2 titre, with complete inactivation reached within a brief incubation [20,21]. NP-40 has shown full inactivation across the same concentration range [20,21]. Tween 20 has not proven effective at inactivating SARS-CoV-2 even at 0.5% [20]. Adding proteinase K to the present buffer would likely strengthen its viral-inactivation profile, but the Triton X-100 and NP-40 concentrations used here are already documented to inactivate SARS-CoV-2 [20,21]. Cationic and zwitterionic alternatives are described in US Patent 10,676,785, where two detergents (designated Dt1 and Dt2) are claimed to enhance polymerase stability and amplification efficiency at lower concentrations than NP-40 or Tween 20, while addressing reduced amplification efficiency and non-specific product formation. Whether such detergents would integrate cleanly with the present one-step workflow, and at what concentrations, has not been tested here.

Sodium azide was tolerated up to 0.1% in the assays reported here, but compatibility is polymerase- and buffer-dependent [23]. The mechanism is direct interference with DNA-polymerase catalytic activity, with effects ranging from loss of sensitivity to outright false negatives [23]. Inhibition can be attenuated through buffer modifications. Raising the buffer concentration is one option. ProClin 300 represents a different preservative strategy. Effective at concentrations of 0.01–0.05%, with minimal enzymatic interference, broad-spectrum antimicrobial activity, and pH stability, ProClin 300 is more compatible with PCR enzymes than sodium azide. Cost works in the opposite direction: ProClin 300 is sold at a premium, while sodium azide is the cheaper, well-established option for molecular-biology buffers. Two routes are therefore available to users adopting the buffer with other enzyme systems: direct retesting of azide tolerance in their specific reaction, or substitution with ProClin 300 at the higher reagent cost in return for broader compatibility.

Finally, the present validation is bounded in scope. The targets tested were UBC and the SARS-CoV-2 RdRP/N panel; the matrices were SaOS-2 cells, MCF7 cells, PBMCs, and nasal and throat swabs. Application to other matrices, especially tissue homogenates and small-RNA workflows where extraction protocol is known to drive divergent outcomes for RNA recovery and sequencing accuracy, and to other reverse-transcriptase or Taq combinations will need independent optimization. Broader validation across food samples, additional clinical matrices, and a wider panel of control nucleic acids is the natural next step toward establishing universal reproducibility.

## 5. Conclusions

This study describes the development and validation of a defined Direct Cell-to-PCR Lysis Buffer for RNA-extraction-free one-step RT-qPCR. The optimized non-ionic detergent formulation, containing Tween 20, Triton X-100, and NP-40, was compatible with RT-qPCR and avoided the complete amplification inhibition observed with ionic detergents. Across cultured cells, PBMCs, and clinical swab specimens, the method produced reproducible amplification with modest Ct offsets compared with column-purified RNA.

The buffer supported UBC detection from single-cell-equivalent input, detected plasmid templates down to approximately 100 copies per reaction, and was compatible with a multiplex SARS-CoV-2 RT-qPCR assay targeting RdRP, N, and RPP30. Sodium azide was tolerated up to 0.1% under the tested conditions. Overall, this formulation provides a rapid and low-cost alternative to column-based RNA extraction that is readily amenable to scale-up for low-input gene-expression analysis and selected molecular diagnostic workflows. Further validation in additional sample matrices, enzyme systems, and target panels will be required before broader diagnostic implementation.

## Figures and Tables

**Table 1:** Primer and probe sequences used for SARS-CoV-2 and human control targets.

Gene	Component	Sequence (5′→3′)
*RdRP*	Forward Primer	GTGAAATGGTCATGTGTGGCGG
	Probe	FAM-CAGGTGGAACCTCATCAGGAGATGC-BHQ1
	Reverse Primer	CAAATGTTAAAAACACTATTAGCATA
*N*	Forward Primer	CTGCAGATTTGGATGATTTCTCC
	Probe	VIC-5′-ATTGCAACAATCCATGAGCAGTGCTGACTC-3′-BHQ1
	Reverse Primer	CCTTGTGTGGTCTGCATGAGTTTAG
*RPP30*	Forward Primer	AGATTTGGACCTGCGAGCG
	Probe	5′Cy5-TTCTGACCTGAAGGCTCTGCGC-BHQ-2
	Reverse Primer	GAGCGGCTGTCTCCACAAGT

**Table 2:** Compatibility of ionic and non-ionic detergents with UBC RT-qPCR.

Detergent (0.025%)	Ct
Control—No detergent	18.59
SDS	Undetermined
NP-40	19.71
NP-9	19.33
Sodium deoxycholate	Undetermined
Sarkosyl	Undetermined
Tween^®^ 20	18.98
Triton™ X-100	18.57

Ct values for UBC RT-qPCR using purified RNA isolated from MCF7 cells in the presence of 0.025% ionic or non-ionic detergents. “Undetermined” indicates no amplification within 40 cycles.

**Table 3:** Titration of non-ionic detergents for RT-qPCR compatibility.

Concentration	Triton™ X-100 (Ct)	Tween^®^ 20 (Ct)	NP-40 (Ct)
0	17.47	17.47	17.47
0.5%	17.51	17.69	17.57
0.3%	17.76	17.49	17.51
0.2%	17.70	17.59	17.27
0.1%	17.47	17.16	17.32
0.05%	17.81	17.46	17.36
0.025%	17.58	17.38	17.26

Ct values for UBC RT-qPCR using purified RNA isolated from MCF7 cells with varying concentrations of Triton X-100, Tween-20, and NP-40.

**Table 4:** Comparison of Direct Cell-to-PCR lysis buffer and column-based RNA isolation across sample types. Ct offsets between direct lysates and column-purified RNA were significant for every matrix (unpaired two-sample *t*-test on technical triplicates: SaOS-2, *p* = 0.0034; MCF7, *p* = 0.0095; PBMC, *p* = 0.0213; throat swab, *p* = 0.0008; nasal swab, *p* = 0.0002).

Sample Type	PureLink Isolated RNA, Ct Mean ± SEM	Direct Cell-to-PCR Lysate, Ct Mean ± SEM	Ct Offset, Direct Minus PureLink
SaOS-2 cells	16.53 ± 0.09	17.53 ± 0.12	1.00
MCF7 cells	18.86 ± 0.03	19.47 ± 0.08	0.61
PBMCs	22.51 ± 0.10	23.18 ± 0.14	0.67
Throat swab	22.94 ± 0.07	25.79 ± 0.16	2.85
Nasal swab	24.58 ± 0.10	27.04 ± 0.05	2.46

Values represent UBC RT-qPCR Ct means ± SEM from three technical replicates per condition. Ct offset was calculated as the mean Ct of Direct Cell-to-PCR lysate minus the mean Ct of PureLink isolated RNA.

**Table 5:** Effect of sample input volume on Direct Cell-to-PCR RT-qPCR performance.

Template Input Volume	PureLink Isolated RNA, Ct Mean ± SEM	Direct Cell-to-PCR Lysate, Ct Mean ± SEM	Ct Offset, Direct Minus PureLink
2.5 µL	15.35 ± 0.15	15.79 ± 0.03	0.44
5.0 µL	14.22 ± 0.13	16.07 ± 0.45	1.85
7.5 µL	14.85 ± 0.23	15.65 ± 0.24	0.80
10 µL	14.51 ± 0.53	16.78 ± 0.24	2.27

Values represent UBC RT-qPCR Ct means ± SEM from three technical replicates per condition using different input volumes of isolated RNA or Direct Cell-to-PCR lysate in a final reaction volume of 20 µL. Ct offset was calculated as the mean Ct of Direct Cell-to-PCR lysate minus the mean Ct of PureLink isolated RNA. Two-way ANOVA showed a highly significant difference between methods (*p* < 0.0001), no significant main effect of input volume (*p* = 0.29), and a significant method × volume interaction (*p* = 0.021).

**Table 6:** Effect of Taq polymerase concentration on RT-qPCR using Direct Cell-to-PCR Lysis Buffer.

Taq Polymerase Concentration	PureLink Isolated RNA, Ct Mean ± SEM	Direct Cell-to-PCR Lysate, Ct Mean ± SEM	Ct Offset, Direct Minus PureLink
0.3125 units	14.26 ± 0.04	17.46 ± 0.02	3.20
0.625 units	14.19 ± 0.12	17.45 ± 0.09	3.26
1.25 units	14.36 ± 0.32	17.12 ± 0.10	2.76
2.5 units	13.90 ± 0.10	16.95 ± 0.19	3.05

Values represent UBC RT-qPCR Ct means ± SEM from three technical replicates per condition using varying Taq polymerase concentrations. Ct offset was calculated as the mean Ct of Direct Cell-to-PCR lysate minus the mean Ct of PureLink isolated RNA. Two-way ANOVA showed a highly significant difference between methods (*p* < 0.0001), a small effect of Taq-polymerase concentration (*p* = 0.044), and no significant method × concentration interaction (*p* = 0.38), indicating a consistent offset across Taq concentrations.

**Table 7:** Detection of UBC expression from varying SaOS-2 cell numbers using Direct Cell-to-PCR Lysis Buffer.

SaOS-2 Cells per Reaction	UBC Ct, Mean ± SEM	ΔCt Relative to 5000 Cells
5000	13.32 ± 0.10	0.00
2500	14.68 ± 0.09	1.36
1000	15.60 ± 0.05	2.28
500	16.11 ± 0.03	2.79
250	17.86 ± 0.15	4.54
100	19.24 ± 0.54	5.92
50	19.88 ± 0.18	6.56
10	22.59 ± 0.71	9.27
1	25.26 ± 0.11	11.94

Values represent UBC RT-qPCR Ct means ± SEM from three technical replicates per cell input using Direct Cell-to-PCR lysates. ΔCt was calculated relative to the mean Ct value obtained from 5000 SaOS to 2 cells per reaction. Linear regression of Ct on log10(cell number) gave a slope of −3.27, R^2^ = 0.994, and an amplification efficiency of 102%.

**Table 8:** Analytical sensitivity of UBC detection across varying plasmid copy numbers.

Plasmid Copies per Reaction	UBC Ct, Mean ± SEM	Detection Status
1,000,000	17.41 ± 0.06	Detected
100,000	21.16 ± 0.07	Detected
10,000	24.63 ± 0.15	Detected
1000	28.05 ± 0.11	Detected
100	33.49 ± 0.76	Detected
10	Undetermined	Not detected
1	Undetermined	Not detected

Values represent qPCR Ct means ± SEM from three technical replicates per plasmid copy number. “Undetermined” indicates no amplification within 40 cycles. Linear regression of Ct on log10 (plasmid copies) over the detected range (100 to 10^6^ copies) gave a slope of −3.91, R^2^ = 0.991, and an amplification efficiency of 80%.

**Table 9:** Effect of sodium azide concentration on multiplex SARS-CoV-2 RT-qPCR performance.

Target Gene	Sodium Azide (%)	Ct Value (Mean ± SEM)
*RdRP*	0	27.71 ± 0.60
	0.01	27.21 ± 0.44
	0.03	27.50 ± 0.41
	0.05	28.03 ± 0.48
	0.10	28.21 ± 0.96
*N*	0	27.88 ± 0.26
	0.01	27.14 ± 0.19
	0.03	27.27 ± 0.12
	0.05	27.79 ± 0.05
	0.10	28.24 ± 0.23
*RPP30*	0	28.89 ± 0.37
	0.01	28.43 ± 0.05
	0.03	28.58 ± 0.08
	0.05	29.35 ± 0.23
	0.10	29.40 ± 0.24

Mean Ct values ± SEM are shown for RdRP, N, and RPP30 targets in the ScriptTaq COVID PCR assay following exposure to increasing sodium azide concentrations ranging from 0% to 0.1%. Values represent three technical replicates (*n* = 3).

**Table 10:** Comparison of the Direct Cell-to-PCR Lysis Buffer with established direct-lysis RT-qPCR methods.

Feature	Present Study	Viet-Phuong Le et al. [8]	Smyrlaki et al. [11]
Lysis chemistry	Three non-ionic detergents (0.3% Tween 20, 0.1% Triton X-100, 0.1% NP-40)	Single non-ionic detergent (IGEPAL CA-630, octylphenol ethoxylate) with bovine serum albumin	Single detergent at high concentration (5% Triton X-100 or 10% Tween 20)
Heat step	None; room temperature	None	Heat-inactivation route (95 °C, 5 min); detergent route without heat
Added protein	None	Bovine serum albumin	None
Ct offset vs. column-purified RNA	0.6 to 2.85 cycles	Approximately 1 cycle earlier than column extraction	Approximately 6.7 cycles (median, heat route)
Reported detection limit	Single-cell-equivalent; ~100 plasmid copies per reaction	As few as ten cells	Clinical-specimen (diagnostic) scale
Diagnostic performance	Compatible with multiplex SARS-CoV-2 assay (RdRP, N, RPP30)	Gene-expression focus	98.8% accuracy, 96% sensitivity, 99.8% specificity
Hands-on time	Minutes; no binding, wash, or elution	Minutes	Minutes; heat step adds a hold
Primary application	Low-input gene expression and selected diagnostics	Low-input gene expression	Large-scale viral diagnostics

## Data Availability

All data are included in this manuscript.
